# Case report of C5 palsy after C3-C6 posterior decompression and instrumented fusion in a patient undergoing inpatient rehabilitation

**DOI:** 10.1051/bmdcn/2018080320

**Published:** 2018-08-24

**Authors:** Tze Chao Wee, Jennifer O’Riordan

**Affiliations:** 1 Changi General Hospital 2 Simei Street 3 Singapore 529889

**Keywords:** C5 palsy, Cervical spine, Cervical decompression

## Abstract

We report on a patient who had neurological deterioration attributed to C5 palsy post C3-C6 posterior decompression and instrumented fusion. A 60-year old man was admitted after a fall from an electric scooter. MRI of the cervical spine confirmed severe cervical spondylosis causing cord compression at C4/5 with associated cord oedema. He underwent posterior cervical decompressive surgery, and he remained neurologically stable post operatively. However, he subsequently developed acute left upper limb weakness limited to the C5 myotome 1 week after surgery whilst undergoing inpatient rehabilitation. A repeat MRI of the cervical spine did not reveal any new changes that may explain his symptoms. He was started on intravenous dexamethasone. C5 palsy after cervical decompressive surgery is not uncommon. There is no specific evidence-based treatment and it carries a generally good prognosis. The aim of this case report is to highlight this complication and raise awareness amongst physicians.

## 1. Introduction

Cervical spondylosis and cervical myelopathy are fairly common in older adults. Decompressive surgery, with or without stabilization, is often indicated for patients with neurological compromise or progressive neurological deterioration. Although C5 palsy is a known complication after cervical spine decompression surgery and has been previously reported in the orthopaedic literature, it is seldom reported in the general medical literature. We report on a case of C5 palsy post C3-C6 posterior decompression and instrumented fusion, including functional outcome post rehabilitation.

## 2. Case Report

A 60-year old man was admitted to the hospital after falling off an electric scooter. He fell on outstretched hands and knees and did not sustain a head injury. He immediately noticed loss of sensation in all four limbs and the abdomen.

On admission to the emergency department, he did not have motor or sensory (light touch or pain) deficits on physical examination. Deep tendon reflexes were intact. Deep anal pressure was intact. Anal tone was preserved with normal voluntary anal contraction. Computed tomography (CT) brain at that time did not show any evidence of fracture or haemorrhage.

However, on review 2 hours later, wrist flexion and extension were noted to have dropped to a power of 2/5, and his grip 3/5 based on the Medical Research Council (MRC) manual muscle testing scale. He was also complaining of a tingling sensation throughout his body. Anal tone and sensation remained normal. The neurological level of injury was C5 ASIA Impairment Scale (AIS) D based on the International Standards for Neurological Classification of Spinal Cord Injury. Please see Table 1. CT cervical spine showed multilevel cervical spondylosis with marginal osteophytosis and uncovertebral hypertrophy. There was mild central canal stenosis noted at C6/7 and bilateral exit foramina narrowing.

**Table 1:** Timeline and neurological assessment.

	2hrs in ED		Day 2		Day 3		Day 5 (Pre-op)		Day 6 POD1		Day 7 POD2		Day 13 POD8		Day 20 POD15		Day 35 POD30		Day 150	
	
FIM											77		75		86		113		124
	R	L	R	L	R	L	R	L	R	L	R	L	R	L	R	L	R	L	R	L
**Motor**																				
C5	5	5	5	4	4	4	4	4	5	4	5	4	5	1	5	1	5	1	5	3
C6	5	2	5	3	4	5	4	5	5	4	5	4	5	4	5	4	5	4	5	5
C7	5	2	5	4	5	5	5	5	5	4	5	3	5	4	5	4	5	4	5	5
C8	5	3	5	3	5	4	5	4	5	4	5	3	5	4	5	4	5	4	5	5
T1	5	3	5	3	4	4	4	4	4	3	5	3	5	5	5	4	5	4	5	5
L2 to S1	5	5	5	5	5	5	5	5	5	5	5	5	5	5	5	5	5	5	5	5
**Sensation (light touch and pin prick)**																				
C2 to C4	2	2	2	2	2	2	2	2	2	2	2	2	2	2	2	2	2	2	2	2
C5	2	2	2	1	2	2	2	1	2	2	2	2	2	1	2	1	2	1	2	1
C6	2	2	2	2	2	1	2	1	2	2	2	1	2	1	2	1	2	1	2	1
C7	2	2	2	2	2	1	2	1	2	1	2	1	2	1	2	1	2	1	2	1
C8	2	2	2	2	2	1	2	1	1	2	2	1	2	1	2	2	2	1	1	1
T1	2	2	2	2	2	1	2	1	1	2	2	1	2	1	2	2	2	1	1	1
T2 to S2	2	2	2	2	2	2	1	1	2	2	2	2	2	2	2	2	2	2	2	2

FIM: Functional Independence Measure.

He was admitted for observation. Magnetic resonance imaging (MRI) of the cervical spine confirmed the presence of severe cervical spondylosis causing cord compression at C4/5 with associated cord oedema. He was kept in a hard Aspen collar. His symptoms fluctuated minimally over the next few days and his neurological level of injury was C4 AIS D prior to surgery. He underwent C3-C6 posterior decompression and instrumented fusion to stabilise the spine and to prevent further deterioration.There were no intraoperative complications and neuromonitoring of both the motor evoked potentials and somatosensory evoked potentials remained stable throughout the procedure. The cord was well decompressed and noted to be pulsating well. He was neurologically stable post-operatively. He was started on intravenous dexamethasone 4 mg three times a day post operatively and subsequently weaned.

He was admitted to the rehabilitation ward on the second post-operative day. Admission neurological findings can be found in Table 1. His admission Functional Independence Measure (FIM) score was 77. FIM score is a measure of impairments with scores from 18 to 126. A lower score indicates a higher degree of functional impairment. His neurological level of injury remained at C4 AIS D.

Motor power deteriorated on post-operative day 8 with shoulder abduction and elbow flexion both dropping to 1 on the MRC scale. He remained a C4 AIS D. This was accompanied by a drop in FIM score to 75 (which is not clinically significant). An urgent MRI spine showed post-surgical changes without epidural collection or haematoma compressing the cord. There were no nerve root impingement or hardware complications evident on MRI.

The spinal surgeons were consulted and agreed he had post cervical decompression C5 palsy based on clinical examination. Electrophysiological studies were not performed. Dexamethasone was switched from the oral to intravenous route to be given at 4 mg three times a day for 2 days (had been 2 mg twice a day per orally). Subsequently, dexamethasone was switched to the oral route and tapered to 4 mg twice a day for 5 days, then 2 mg twice a day for two days and then 2 mg daily for 2 days before stopping. He continued with inpatient rehabilitation. On discharge from the rehabilitation ward (18 days after his deterioration) the weakness related to his C5 palsy had not improved.

He continued rehabilitation at a subacute facility and was seen in the outpatient clinic at 5 months post injury. He had made further improvement in upper limb strength but some patchy sensory impairment of his left upper limb persisted. He was independent at home despite his reduced hand function and ambulated with no aid. His FIM score had improved to 124 although the neurological level of injury remained at C4 AIS D.

## 3. Discussion

Patients may undergo inpatient rehabilitation after cervical decompressive surgeries, particularly if they have neurological compromise prior to the surgery. With increasing demand for hospital beds, there is increasing pressure for early discharge. Neurological deterioration attributed to C5 palsy may occur in the postoperative period, hence it is vital for both physicians practicing in the hospital and community to be familiar with this condition. In the context of postoperative neurological deterioration, other differential diagnosis should also be considered. Development of an epidural collection such as a haematoma or abscess at the surgical site will need to be excluded.

There is no standard definition of C5 palsy in the literature. Patients will usually present with new weakness in shoulder abduction and elbow flexion/supination as the deltoids and biceps brachii are affected. They may also have sensory deficits or pain in the distribution of the C5 dermatome.[1]

The incidence of C5 palsy varies widely across different studies, surgical approaches and surgical techniques. A narrative review showed that the incidence of isolated C5 palsy varied between 0% and 30%.[2] A systematic review by Gu *et al.* showed that the pooled incidence of C5 palsy after posterior decompression was 5.8%.[3] In another systematic review, the overall pooled prevalence of C5 palsy was 5.3% and patients after posterior cervical surgery (5.8%) had a slightly higher prevalence than those after anterior surgery (5.2%).[4] Bilateral C5 palsy, though rare, has also been reported with an overall incidence of 0.37%, representing 8.8% of all postoperative C5 palsy.[5]

In one of the largest known studies of postoperative C5 palsy to date, the time to initial onset of symptoms ranged from immediately post-operatively to 14 days post-operatively. Slightly more than half of the patients experienced delayed onset (>24 hours post-operatively) C5 palsy.[6] However, in an earlier retrospective review, some patients presented as late as 2 months post operatively.[3] In a retrospective study, Imagama *et al.* found that weakness was noted after a mean of 4.7 (range of 1 to 28) days post-operatively and the mean MMT score at the onset of paralysis was 1.6 for the deltoid and 2.8 for the biceps brachii. They also found that patients with more severe motor deficits at presentation were less likely to have a complete recovery. [1]

The etiology of C5 palsy remains unclear and various theories have been proposed. These include C5 tethering after posterior decompression, reperfusion injury, impairment of C5 nerve root, potential risk of thermal damage to cervical nerve roots with use of high speed drill.[7-10] One study had shown that posterior laminectomies, multilevel corpectomies and procedures with extensive spinal cord shift were associated with C5 palsy. [11] Some studies had found that presence of intramedullary linear high intensity areas on both preoperative and postoperative T2-weighted MRIs were seen more at the level of paralysis but the association of these observations and the occurrence of C5 palsy were not consistent.[1,12-14].

There is no specific treatment for C5 palsy. There is no high- quality evidence for pharmacotherapy, including dexamethasone or other corticosteroids. Conservative management is the usual approach. A case report of bilateral C5 palsy has been described with the restarting of a steroid taper- similar to what was described in our case.[4] The patient’s symptoms persisted for 3 months, before making slow improvement at 6 months and progressing to complete resolution by 1 year.

The prognosis of C5 palsy is generally good. In one study, 54.2% of patients reported complete resolution of symptoms, 25.4% improved with residual symptoms while 17% did not recover. In that study, 44.4%, 59.3% and 95.6% of patients who reported complete resolution of symptoms did so by 3 months, 6 months and 12 months respectively. Nassr *et al.* reported in another study that 71.4% of patients reached maximal improvement within 6 months.[15]

In conclusion, we would like to highlight a case of C5 palsy post posterior decompression and fusion from C3 to C6 levels, which occurred while the patient was undergoing inpatient rehabilitation, and his subsequent favourable clinical course with conservative management. Although C5 palsy is not considered rare and widely published in the orthopaedics literature, its relative absence in the general medical literature prompts a need to remind all physicians of its typical presentation and clinical course.

## Conflicts of interest

The authors declare no conflict of interest.
